# Using a graph-based image segmentation algorithm for remote vital sign estimation and monitoring

**DOI:** 10.1038/s41598-022-19198-1

**Published:** 2022-09-07

**Authors:** Xingyu Yang, Zijian Zhang, Yi Huang, Yalin Zheng, Yaochun Shen

**Affiliations:** 1 Department of Electrical Engineering and Electronics, University of Liverpool, Liverpool, L69 3GJ UK; 2 Department of Eye and Vision Science, University of Liverpool, Liverpool, L7 8TX UK

**Keywords:** Health care, Engineering, Mathematics and computing

## Abstract

Reliable and contactless measurements of vital signs, such as respiration and heart rate, are still unmet needs in clinical and home settings. Mm-wave radar and video-based technologies are promising, but currently, the signal processing-based vital sign extraction methods are prone to body motion disruptions or illumination variations in the surrounding environment. Here we propose an image segmentation-based method to extract vital signs from the recorded video and mm-wave radar signals. The proposed method analyses time–frequency spectrograms obtained from Short-Time Fourier Transform rather than individual time-domain signals. This leads to much-improved robustness and accuracy of the heart rate and respiration rate extraction over existing methods. The experiments were conducted under pre- and post-exercise conditions and were repeated on multiple individuals. The results are evaluated by using four metrics against the gold standard contact-based measurements. Significant improvements were observed in terms of precision, accuracy, and stability. The performance was reflected by achieving an averaged Pearson correlation coefficient (PCC) of 93.8% on multiple subjects. We believe that the proposed estimation method will help address the needs for the increasingly popular remote cardiovascular sensing and diagnosing posed by Covid-19.

## Introduction

Vital signs are measurements of the essential physiological functions of a human body. The most routinely checked vital signs include heart rate (HR), breathing rate (BR), body temperature and blood pressure. Vital sign data is also one of the primary data collected for telehealth services for remote health monitoring^1^. Conventionally, these vital sign data are measured via contact-based technologies, such as electrocardiography (ECG) and pulse oximetry^2^. As the Covid-19 pandemic is forcing a digital transformation of the healthcare industry, contactless and remote monitoring technologies are replacing the predominant position of contact-based devices in telehealth and clinical settings^3,4^.

Vital signs are normally estimated from either detecting the chest and heartbeat displacements or face blood volume pulse (BVP) signal. Previous studies have explored the feasibility of remote heart rate monitoring using frequency modulated continuous wave (FMCW) radars. Time-varying filters, phase drift reduction, and motion suppression algorithms are reported to be effective when using FMCW with various starting frequencies^5–8^. Recorded face and chest videos have also been used to obtain cardiac pulse measurements. As cardio-vascular activities can cause subtle intensity variance on the human face, a photoplethysmogram (PPG) can be generated to compute the BVP and eventually the HR^7,8^. Both visible and near-infrared (NIR) spectrums have been studied for different applications^9,10^. For both radar and video monitoring modalities, the most widely used method for estimating vital signs is finding the most prominent spectral magnitude of the resultant Fourier transform of the measured time-domain signal.

However, remote vital sign measurements suffer from accuracy and reliability issues. FMCW radar is intrinsically affected by phase randomness, harmonics, and interference from the antenna, while the recorded videos are susceptible to environmental illumination variance. Both techniques are highly susceptible to motion disruptions when measuring or monitoring in real-life scenarios^6,7,11,12^. The disruptions primarily limit the wide implementation of remote vital signs monitoring technologies in clinical settings. In past studies, test subjects were required to calmly sit or lie down to avoid motion disruptions or drastic HR/BR changes^13,14^. Nevertheless, patients are likely to experience stress in clinical settings, which would cause body movements and fluctuation in vital signs measurements. Therefore, vital signs monitoring systems require more clinically practical body movement mitigation. In this work, we will focus on the development of the advanced method for the robust extraction of vital signs from recorded video and radar signals.

Several HR extraction methods have been reported^12–15^. The methods share a common ground of performing signal processing-based extractions on a sequence of individual waveforms. The extraction methods vary from the maximum spectral magnitude, peak counting, and machine learning. Compressive sensing (CS) and discrete wavelet transform (DWT) based algorithms have also been proposed to increase the precision of estimation^5^. However, external disruptions can cause significant losses of signal and accuracy when performing signal processing-based methods on individual waveforms. Deep learning (DL) based extraction methods have also been investigated in extracting vital signs from videos, FMCW radar signals, etc^16,17^. However, due to the limitation of public datasets and accuracy, DL methods remain to be an active field of research. Instead, studies have found DL methods useful in finding the optimal measurement window and region-of-interest (ROI) in real-life situations^18,19^.

Time–frequency analysis (TFA) is a widely applied method in continuous wave radar technology, including micro-Doppler analysis, target recognition, driving behaviour detection, etc^20–24^. Short-time Fourier transform (STFT) is a linear TFA method that intuitively illustrates the variation in signal frequency over a short period of time. Instead of analysing the frequency energy over the entire measurement data, STFT offers time-localised frequency information, which is useful in situations where the frequency components of a signal vary over time. Vital sign estimation is one of the situations in which the local frequency information needs to be extracted. The periodic chest motion of breathing and heartbeats can be visualised by applying the STFT technique with a sliding window to generate a time–frequency spectrogram^25,26^.

In this work, we report an image segmentation-based HR and BR extraction method to improve the measurement accuracy and robustness. Rather than using a single waveform, the proposed method extracts the vital signs from the STFT spectrograms using graph weight and normalised cuts image segmentation^27^. We discovered that the layered structures in the vital sign spectrogram fit the criteria for applying the algorithms. The filtering and sliding window techniques contribute to the continuity of the spectral energy distribution in the spectrogram. This study expanded the scope of retinal layer algorithms to be applied in the field of spectral analysis. An open-source retinal layer segmentation software is adopted for our application^28^.

As a result, the less prominent vital sign signals that are submerged by noise and motion corruption will be highlighted, leading to much-improved measurement robustness and accuracy. This approach is able to perform on both radar and video data. The method is validated against the gold standard, e.g., a commercial contact-based ECG device (Polar H10). The experiment was conducted in a controlled lab environment, with the test subject taking measurements before and after physical exercises. The results of the individual devices and the combined system are cross-compared with the gold standard method. Pearson correlation coefficients (PCC), root-mean-square error (RMSE), area under the curve of success rate (AUC-SR) and coverage are employed as the performance and accuracy indicators. The statistics of the results are visualised by boxplots.

The results demonstrate that the proposed image segmentation method provides considerably improved accuracy and robustness when facing interference and disturbance compared with the conventional spectral magnitude method.

## Methodology

### The FMCW radar based measurement principles

**Figure 1:**
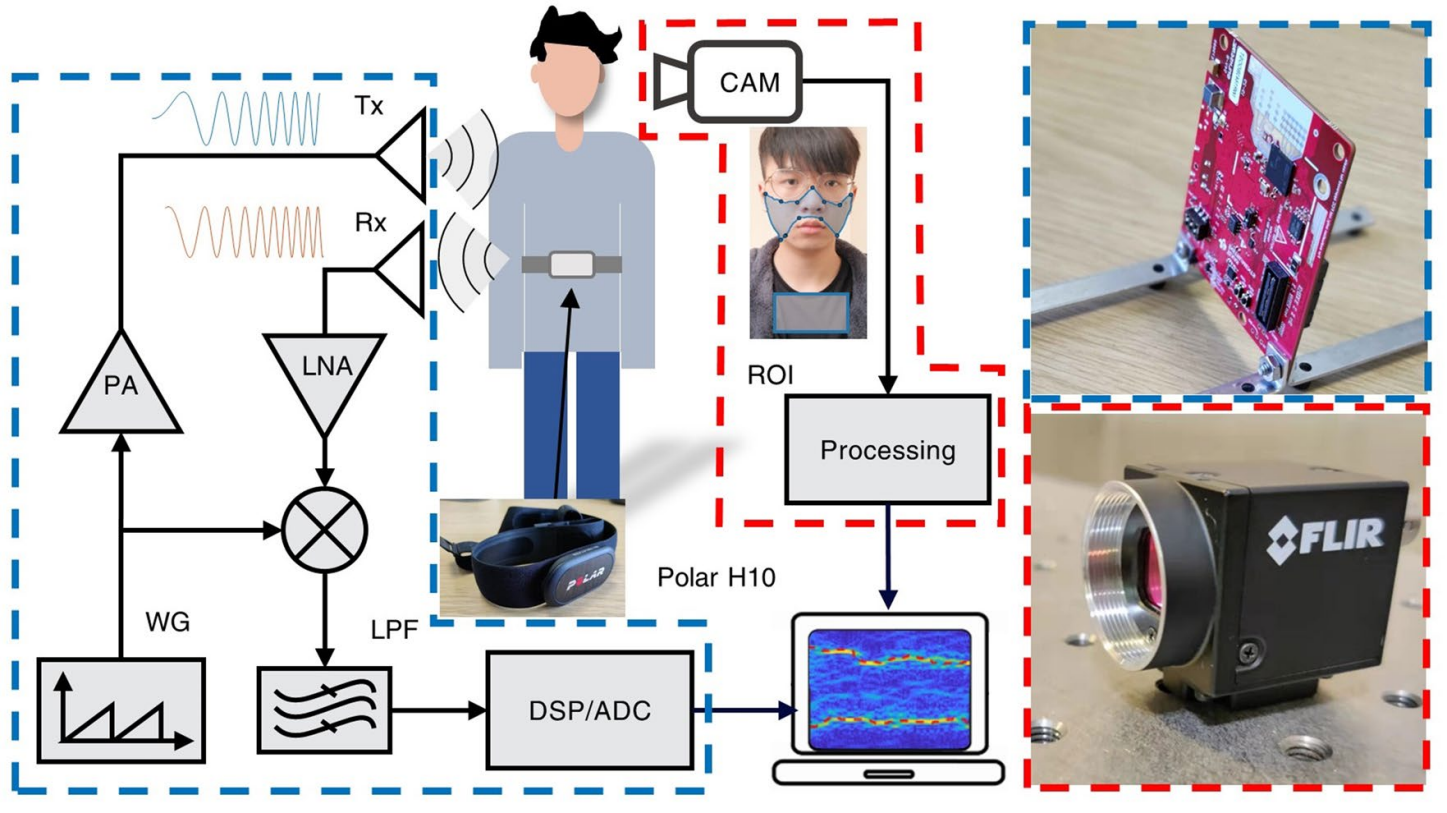
The schematic diagram of the system. WG: waveform generator; PA: pre-amplifier; LNA: low-noise amplifier; LPF: low-pass filter; DSP: digital signal processing unit; ADC: analogue–digital-converter; TX/RX: transmitting/receiving antennas. The blue and red enclosing is the photo of the FMCW radar and the camera used in the experiment, respectively.

Figure 1 shows the system schematic diagram for simultaneous video and radar vital signs monitoring. The FMCW device selected in this experiment was an mm-wave radar (IWR 1843, Texas Instruments), with an operational frequency range of 77–81 GHz. Out of the three transmitting and four receiving antennas equipped on the radar, only the closest spaced pair of transmitting/receiving antennas (TX/RX) was used. Chirps are generated by a waveform generator and transmitted via a self-oscillation circuit, a mixer, and a pre-amplifier for the transmit antenna. The received signals are passed through a low-noise amplifier, a low-pass filter, a digital signal processing unit, and an analogue–digital converter (ADC) for the computer to perform further processing.

The principles of FMCW radar have been explained in detail in multiple research^5–7,11^. The conventional use of the FMCW radar is to perform a range FFT on chirp sets that contain distance information and generate a range bin map. In the application of vital sign measurement, the amplitude of the chest movement is 4 -12 mm, while the amplitude of heart pulses ranges from 0.1 to 0.5 mm^29^. In order to resolve these small-scale movements, the phase shift $\Delta \varphi$ between two consecutive chirp signals will be calculated first. The displacement $\Delta R$ of the object can then be calculated as:

1 $${\Delta }R = \frac{\lambda }{4\pi }{\Delta }\varphi$$

where $\lambda$ is the central wavelength of the radar.

**Figure 2:**
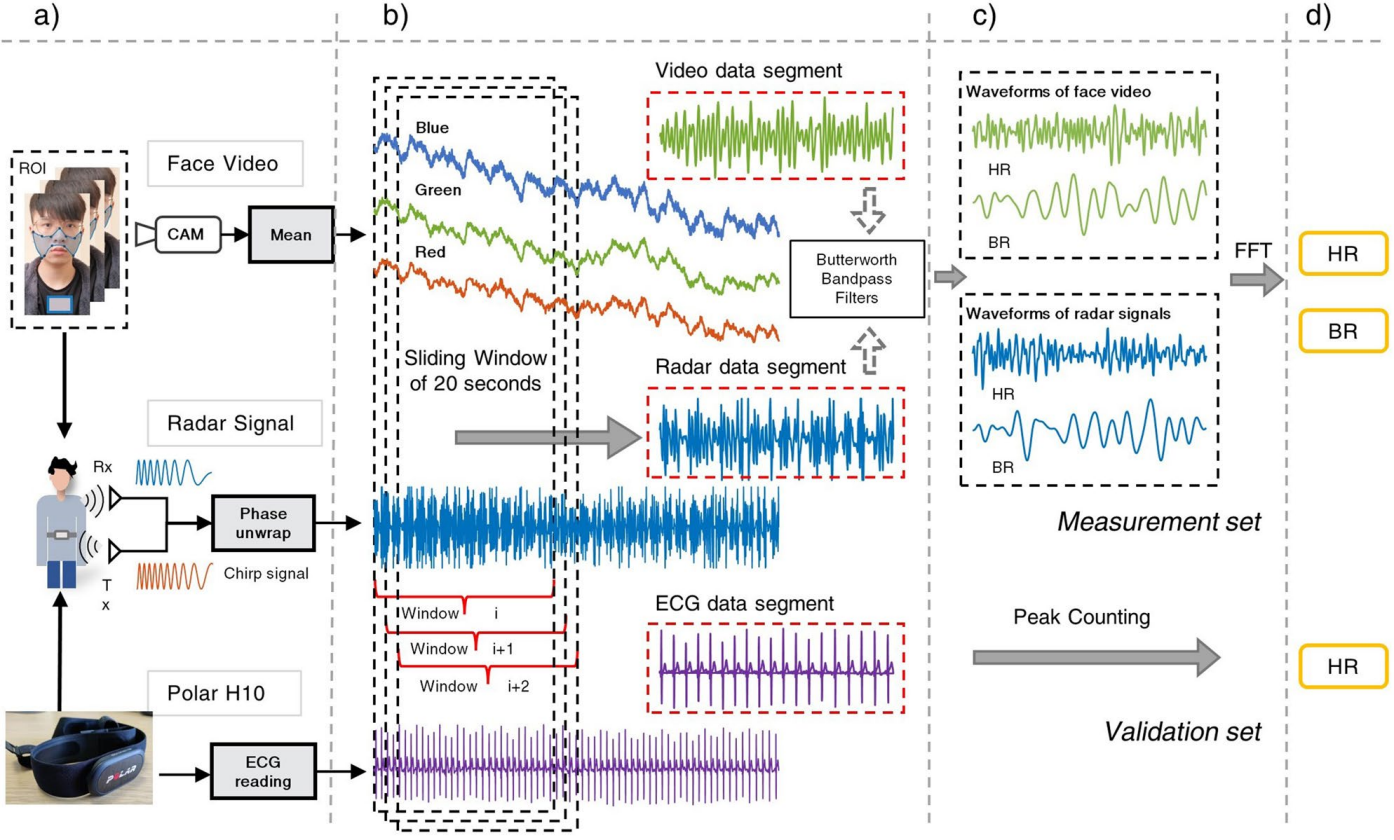
(**a**) The sources for measurement and the initial processing. (**b**) The processed data waveform and the sliding window. (**c**) Filtered waveforms from the remote monitoring systems and the estimation methods. (**d**) The final estimations from the measurement set and validation set.

As shown in Fig. 2a, the primary processing technique involves performing phase unwrapping on the phase term of a set of chirps to obtain the correct phase information. A 20-s sliding window is then applied to compute vital signs second by second, as shown in Fig. 2b. Similar to the previous studies, phase randomness and spike noises are removed by computing the phase difference and energy-based thresholding^6,7^.

Afterwards, a digital fourth-order Butterworth filter is applied in the frequency band of 0.2–0.6 Hz for respiration rate and 1–4 Hz for heart rate extraction, respectively. The resulting waveforms represent the heartbeat and breathing patterns in the data segment, as shown in Fig. 2c.

As the breathing and cardiac cycles tend to be periodical, the vibration frequency can be extracted by applying a second FFT. The data is zero-padded with three times the data size to allow more data points in the resulting spectrum. Conventionally, the largest spectral magnitude of the resultant FFT spectrum corresponds to the HR (heartbeat frequency) and BR (breathing frequency). However, due to spectral noises caused by motion disruptions, the largest magnitude may not always produce the correct heart rate estimation. Here we propose a novel image segmentation-based method to address this issue.

### Video-based measurement principles

Intensity-based face video vital sign extraction stems from photoplethysmography (PPG), a common and low-cost optical technique. Since light is more strongly absorbed by blood than the surrounding tissues, the periodic changes in blood flow can be detected by PPG sensors as changes in the intensity of light^30^. Thus, the subtle change in the light intensity on human skin can be captured by a digital camera. The face video vital sign extraction process can be referred to Fig. 2.

Past study found facial regions around the forehead, cheeks and mouth tend to be more reliable for cardio-vascular pulse signal extraction^31^. Areas close to eyes and mouths are less suitable as they are likely to be affected by facial muscles^14^. Breathing pattern signals are most reliable around the chest area, corresponding to ventilation movements. As shown in Fig. 2a, the cardio-vascular pulses for HR and BR are extracted from a manually selected face ROI and a box ROI on the chest area, respectively.

At first, the spatial average is employed to improve the SNR of the raw signal containing cardio-vascular pulse information and enhance the subtle colour changes^12,32–34^. Then, the pixel values of each colour channel in the selected ROI are averaged for each video frame to overcome sensor and quantisation noise. Unlike the conventional PPG-based devices utilising Near-Infrared light, the blood volumetric variation is reflected in three channels of colour video. Because haemoglobin and oxyhaemoglobin in blood both have higher absorption in the green channel than the red channel^35^, the green channel is selected to deliver the optimal SNR, as shown in Fig. 2b. The same 20-s sliding window is applied.

The obtained signal waveform comprises "AC" components that are synchronous with each heartbeat and respiration. The slowly varying "DC" baseline corresponds to the subtle changes in illumination and head motion, even in a strictly controlled environment. Thus, a detrending filter is required to reduce the low frequencies and non-stationary trends of the raw signal^8,11^. This filter is effectively a high-pass filter with negligible latency. Then, a moving-average filter is applied to remove random noise caused by sudden light intensity changes or motion in the frame sequence.

Finally, the same Butterworth bandpass filter as mentioned in the FMCW radar signal processing section is applied to generate the heart and breathing waveforms, as shown in Fig. 2c. HR and BR oscillations are the most periodic components in their frequency bands. They can be located as the signal with the most prominent power magnitude in the spectrum after applying the FFT. Similar to FMCW radar, the impact of motion disruption and change in illumination causes the peak values of the power amplitude to lack continuity and accuracy in video-based HR and BR measurement.

### Graph-based image segmentation estimation method

The radar and video signals are processed and filtered into 1-D time-domain waveforms of HR and BR oscillation, where the most periodic components indicate the actual beat frequencies. Instead of applying FFT to the entire time-domain waveform to calculate the beat frequency, here, STFT is used to generate time–frequency STFT spectrograms:

2 $$STFT\left\{ {x\left( t \right)} \right\} \equiv X\left( {\tau ,\omega } \right) = \mathop \int \limits_{ - \infty }^{\infty } x\left( t \right)Window \left( {t - \tau } \right)e^{ - i\omega t} dt = \mathop \int \limits_{\tau - B}^{\tau + B} x\left( t \right)e^{ - i\omega t} dt$$

where $x\left(t\right)$ is the time-domain waveform, ω is the angular frequency, and $Window (\tau )$ is the window function. In this study, the sliding window size is set to 20 s; hence the integration limit can be changed to ${\tau}\pm B$, where $B=10s$. The data point spacing of time-domain waveform $\Delta t$ was 0.05 and 0.033 s for FWCM and video data, respectively.

**Figure 3:**
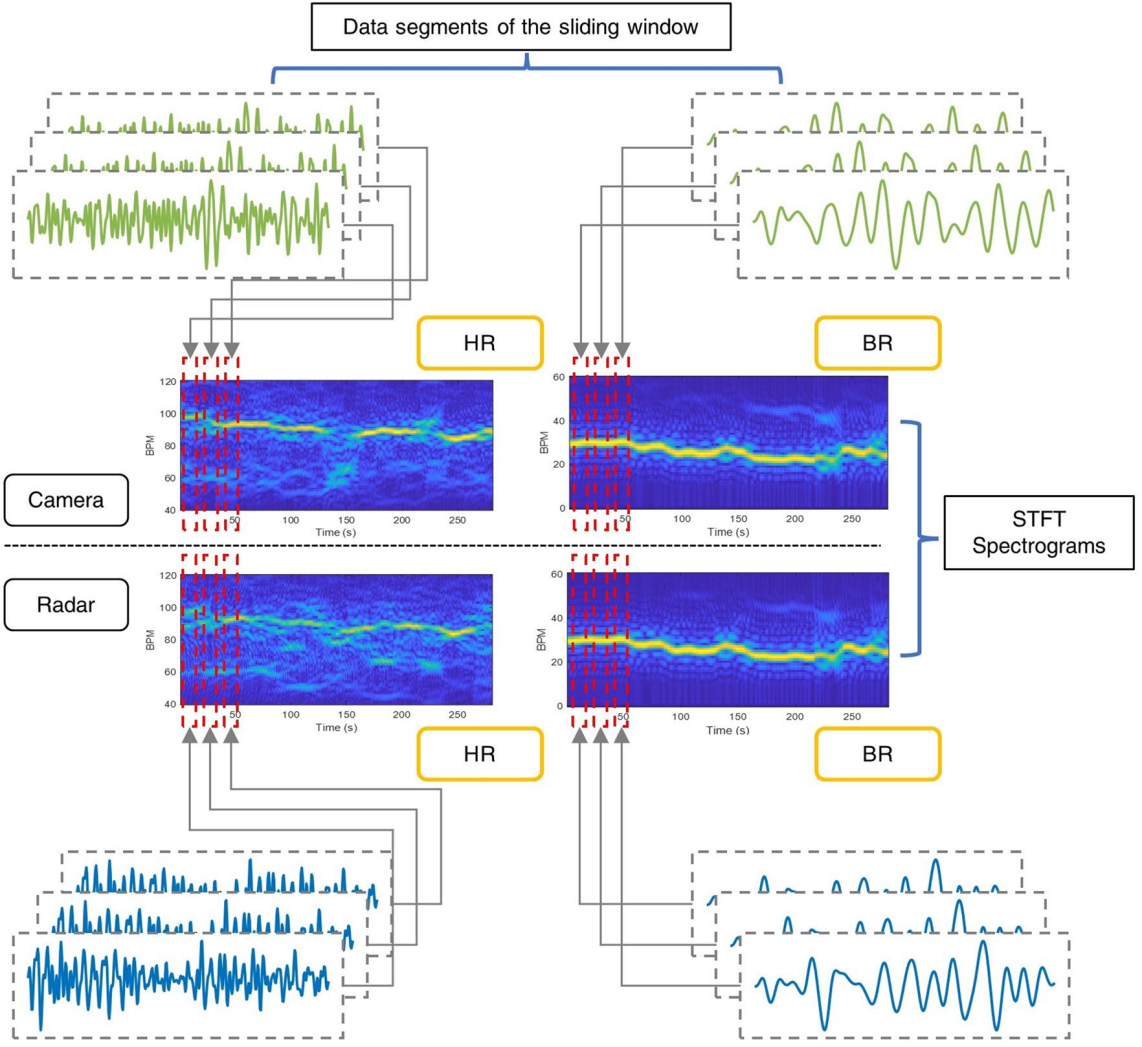
Generating the Short-Time Fourier Transform spectrograms from the 20-s data segments of face video and FMCW radar signals.

By accumulating each frequency domain 1-D signal transversely, the time–frequency spectrogram is generated. After converting the frequency unit from Hz to BPM, the spectra from the data segments form an STFT spectrogram to visualise the vibration signal strength, as shown in Fig. 3. Performing an image segmentation algorithm on the STFT spectrograms will compensate for the disruptions and reduce the impact of external factors compared to the conventional signal processing method.

The estimation method we adopted is graph-based image segmentation^27,28^. The work was originally developed for segmenting the retinal layers in the cross-sectional images from Optical Coherence Tomography (OCT)^27^. The algorithm is generalised for layered structure segmentation, which is an ideal method for extracting the vital signs in STFT spectrograms.

To summarise the segmentation method, each spectrogram is treated as an image, where the pixels are represented by a graph of nodes (or vertices), denoted as ${v}_{i}\in {\varvec{V}}$. The nodes in the graph are connected by edges, denoted as ${e=(v}_{i},{v}_{j})\in {\varvec{E}}$. The graph can then be constructed as ${\varvec{G}}=\left({\varvec{V}},{\varvec{E}}\right)$.

When cutting a graph into segments, a route between the start and the end nodes needs to be created by assigning weights to edges. In literature, graph weights are often represented by the geometric distance and intensity difference of the graph nodes^36^. Considering a mono-colour brightness image, the weight calculation expressed in literature^36^ is:

3 $$w_{ij} = e^{{\frac{{ - \parallel {\varvec{I}}\left( i \right) - {\varvec{I}}\left( j \right)\parallel_{2}^{2} }}{{\sigma_{I} }}}} *\left\{ \begin{gathered} e^{{\frac{{ - \parallel {\varvec{P}}\left( i \right) - {\varvec{P}}\left( j \right)\parallel_{2}^{2} }}{{\sigma_{P} }}}} \quad if\; {\varvec{P}}\left( i \right) - {\varvec{P}}\left( j \right)_{2} < r \hfill \\ 0\quad \quad \quad \quad \;\;\;\;\;otherwise, \hfill \\ \end{gathered} \right.$$

where ${\varvec{P}}\left(i\right)$ is the spatial location of pixel $i$, and ${\varvec{I}}\left(i\right)$ is the intensity-based vector of $i$. $r$ is a user-defined value for the Euclidean norm boundary.

However, due to zero-padding in FFT and filtering, the transition between the adjacent pixels in the spectrograms is smooth, allowing us to consider the intensity difference in the adjacent nodes only for weight calculations. The non-adjacent nodes will not contribute to the weight assignment in this case. Notably, the intensity of pixels corresponds to the spectral magnitudes. In the spectrograms, the signals to be extracted are layer-like and primarily horizontal. Therefore, the difference in intensity ${w}_{xy}$ can be represented by finding the vertical gradients of the image:

4 $$w_{ij} = 2 - \left( {g_{i} + g_{j} } \right) + w_{min}$$

Where ${g}_{i}$ and ${g}_{j}$ are the vertical gradients of the image at node $i$ and $j$, respectively. ${w}_{ij}$ is the weight assigned to node $i$ and $j$, and ${w}_{min}$ is the minimum weight of the graph, added for system stabilisation.

**Figure 4:**
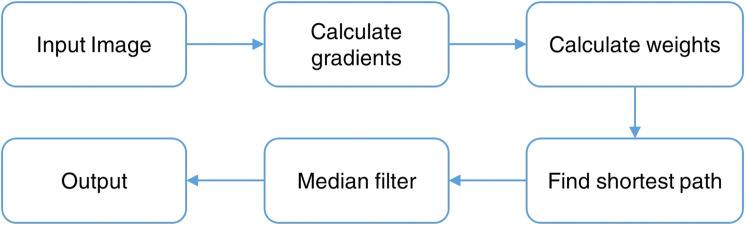
The flowchart of the graph-based image segmentation for layered structures.

An optimal path can be formed if the sum of assigned weights is at the minimum, which, in this case, is determined by using Dijkstra's algorithm^37^. The path is passed through a median filter and can then be considered as the extracted vital sign. Since the spectrograms are likely to consist of a single-layered structure after pre-extraction processing, the search region limitation is not necessary. The segmentation-based method can be used on spectrograms generated by both the radar and the camera to extract vital sign readings. The processing flow is demonstrated in Fig. 4.

### Ethical approval and informed consent

The human image appeared in Fig. 1&2 is a photo of the author Mr Xingyu Yang. Mr Xingyu Yang has given the informed consent for it to be published. The test subjects are fully informed with the purpose of, and methods involved in the study. The subjects have given their informed consents for conducting the experiment and for the data/results to be published. This research was approved by the Committee on Research Ethics of the Dept of Electrical Engineering and Electronics at the University of Liverpool. The devices involved in the study are fully safe for human testing. The experiment protocols and methods follow the safety regulations of and are approved by the Dept of Electrical Engineering and Electronics at the University of Liverpool. The datasets used and/or analysed during the current study are available from the corresponding author upon reasonable request.

## The experiment

### Experimental setup

In this experiment, the chirp duration is set to *T*_*c*_ = 50 µs with an idle time of 7 µs. The available bandwidth of the device is 4 GHz, resulting in a frequency slope *S* = 70 MHz/µs. Each chirp recurs every *T*_*f*_ = 50 ms, which is equivalent to the sampling rate of 20 Hz. A customised Python program was developed in-house to receive and process the FMCW signals in real-time. Face videos are captured by a PointGrey colour camera (Blackfly BFLY-U3-13S2C) with a selected resolution of 640 × 480 pixels. In principle, any camera with sufficient pixel resolution and 30 Hz frame rate can be used for this application^32,34^. The point grey camera is chosen in this study to provide a stable frame rate at 30 Hz to later compare with FMCW measurements and the ground truth. All measurements were performed under a mixed room and natural light illumination at room temperature. The recorded raw data are transmitted to the PC for further processing.

The ground truth was simultaneous ECG measurements obtained using the Polar H10 heart belt as the gold standard. The beats-per-minute (BPM) with respect to time results from three sources were cross-compared for validation.

### The measurements

In the experiments, the FMCW radar and the camera were situated side-by-side at a distance of one meter in front of the test subject. The heart belt was placed around the chest of the subject. The data processing and validation from the three devices are performed offline by in-house software written using MATLAB (2020a). The three devices are configured to be triggered by the in-house developed software. Timestamp alignment is employed to compensate for the delay in starting time, introduced by either hardware initiation or software triggering.

The experiments were designed to test the performance of the proposed extraction method and the integrated system in both post-exercise and pre-exercise conditions. The subject was requested to sit stationary during tests in both conditions. In the former condition, the subject needs to conduct physical exercises prior to the test. Signal losses are expected as the unconscious body and head movements might happen due to post-exercise hyperventilation. In the pre-exercise condition, the subject was requested to refrain from large movements for at least 15 min before testing to reduce unconscious movements.

The test duration was set to 5 min. As the HR and BR signals are sensitive to noise, a 20-s sliding window was implemented to segment data and increase reliability. For comparison, five sets of measurements were conducted on each exercise condition on the same subject. The experiment was then repeated in the same environment with three individuals across two different sessions. The participants were required to remain calmly seated in front of the system during the test. No physical exercise was required prior to the test. The participants were measured for two minutes in each session and were asked to wait for a few minutes before starting the next session.

### The operation flow

**Figure 5:**
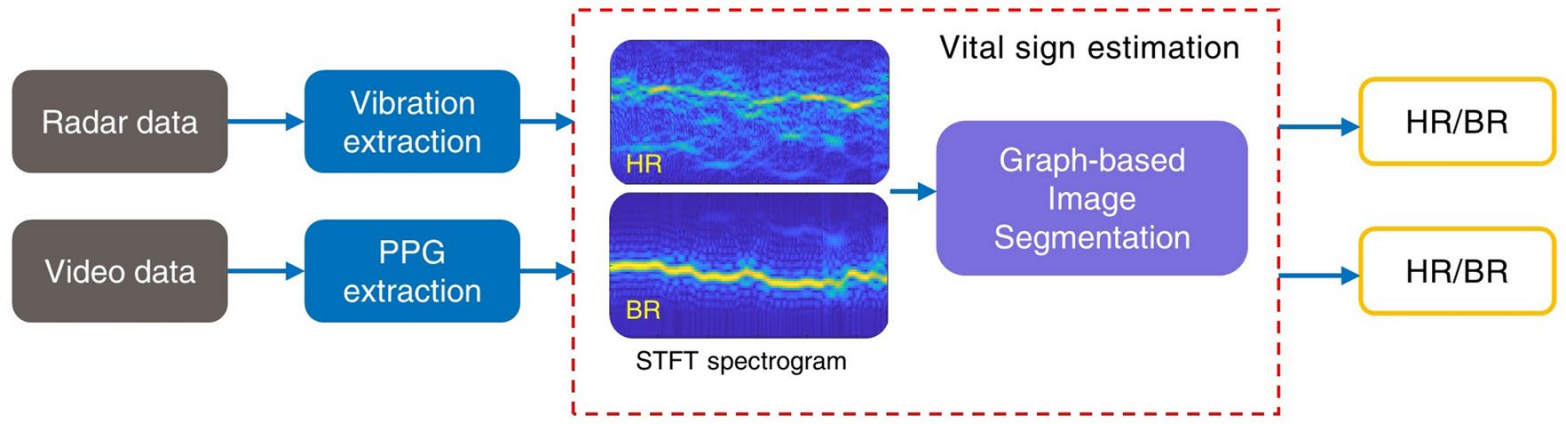
The system operation flow chart for vital signs estimation using data from FMCW radar and face video recording.

The operation flow of the proposed estimation method is shown in Fig. 5. The collected data from the radar and camera recordings can be passed through the same vital sign estimation process. For radar data, the chirp signals are processed to measure the vibration frequency of the test subjects. Heartbeat and respiratory vibrations can be separated by applying bandpass filters with different passbands. The vibration of each time point can be further used to extract the frequency and construct the STFT spectrograms. The graph-based image segmentation is then performed on the constructed spectrograms, which can be considered as images. The optimal paths found by the algorithm are used as the final HR and BR estimation. Similarly, the PPG signals are extracted for video recordings by characterising the intensity variance on the face or chest videos. The frequencies of the emergence of peaks in PPG signals can be used to construct the STFT spectrograms after Fourier transform. The same image segmentation method is performed on the spectrograms, and outputs are considered as the HR and BR estimations.

### The evaluation methods

We employed four evaluation metrics to determine the algorithm and system performance, as proposed in past literature^9,10^. The Pearson Correlation Coefficient is also used to reflect the accuracy of measurements. Each dataset is evaluated under the four metrics.

#### Root mean square error (RMSE)

RMSE is calculated by finding the squared and then averaged difference between the measured data and the corresponding ground truth data. The square root of the average is then taken. As the errors are squared before being averaged, the RMSE gives a relatively high weight to large errors. If RMSE equals 0, it means the perfect fit of two datasets. Therefore, a smaller value of RMSE indicates a more accurate estimation.

#### Area under the curve of the success rate (AUC-SR)

The AUC-SR is found by first calculating the absolute difference between the measured and the ground truth data. Then, the percentage of the data points under the tolerance range (T) can be considered the SR. T is determined to be within [0, 10] bpm. Lastly, AUC is chosen as the quality indicator, where a larger number means a higher success rate. Note that the AUC is normalised by the area under ten and thus varies in [0,1].

#### Coverage at ± 3 bpm

The success rate with T set as three is selected to calculate the measurement coverage. This metric offers a direct view on the percentage of time when the difference between the measured and gold standard HR is within 3 bpm.

#### Pearson correlation coefficient (PCC)

PCC is used as a parameter to assess the similarity between the measured data and ground truth data. The PCC value is between − 1 (strong negative relationship) and + 1 (strong positive relationship). The coefficient R can be expressed as:

5 $$R = \frac{{\mathop \sum \nolimits_{i = 1}^{N} \left( {d_{ai } - \overline{d}_{a} } \right)\left( {d_{bi} - \overline{ d}_{b} } \right)}}{{\sqrt {\mathop \sum \nolimits_{i = 1}^{N} \left( {d_{ai} - \overline{d}_{a} } \right)^{2}\left( {d_{bi } - \overline{ d}_{b} } \right)^{2}} }}$$

where ${d}_{ai}$ and ${d}_{bi}$ are the *i* th elements of the measured and ground truth data, respectively. ${\overline{d} }_{a}$ and ${\overline{d} }_{b}$ are the mean values of the data. $N$ is the number of elements in the dataset.

## Results and discussions

**Figure 6:**
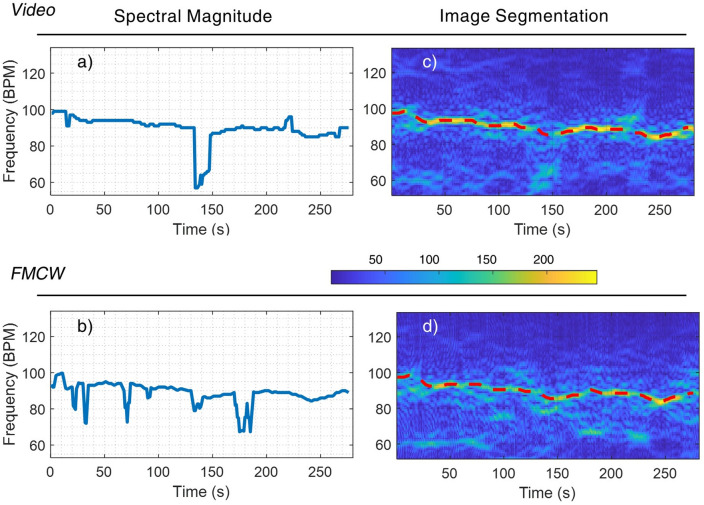
Simultaneous 5-min HR measurements using both the camera and FMCW radar. (**a**) and (**b**): HR estimation using the conventional largest spectral magnitude method. (**c**)–(**d**): The joint STFT spectrograms, where the red dashed lines indicate the HR readings estimated using the image segmentation method.

The data collected from both the camera and radar sources are used to construct the 5-min STFT spectrograms, as shown in Fig. 6. Only 280 s are shown because of the sliding window effect. The spectral magnitude represents the presence of the most substantial frequencies (colour map parula). The noise of interference and motion disruption are visualised by the small-scale magnitude surrounding the main signals. The two columns show the performance of the conventional signal processing method and the proposed image segmentation method, respectively. The red dashed lines in the spectrograms are the estimated HR signals obtained by the proposed image segmentation method. Notably, the spectral magnitude results from Fig. 6a and b are obtained by conventional signal processing method instead of the spectrograms.

**Figure 7:**
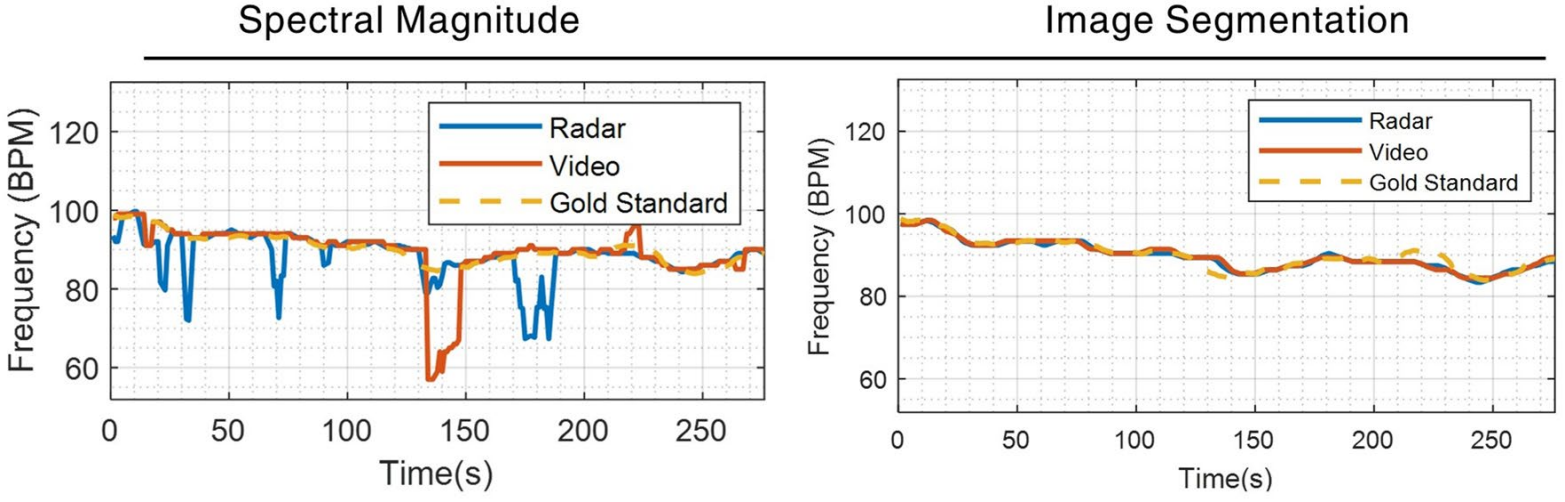
The 5-min HR estimations. Left: the largest spectral magnitude method against the gold standard ECG device reading. Right: the graph-based image segmentation method against the gold standard ECG device reading.

The discontinuities of the spectral magnitude and sudden change of estimation in Fig. 6a and b are mostly caused by failing to distinguish the largest magnitude in the spectrums. This is most likely caused by motion and environmental disruptions. The two methods are further verified by comparing them with the time-traced results from the gold-standard ECG device, as illustrated in Fig. 7. This provides an intuitive visual comparison of the performance of the proposed and conventional approaches. Multiple drops and mismatches are observed in the spectral magnitude method when comparing the processed signals from radar and video sources to the gold standard. In contrast, the image segmentation method generates visually highly similar results from two devices. The results are also close matches to the gold standard measurements.

**Figure 8:**
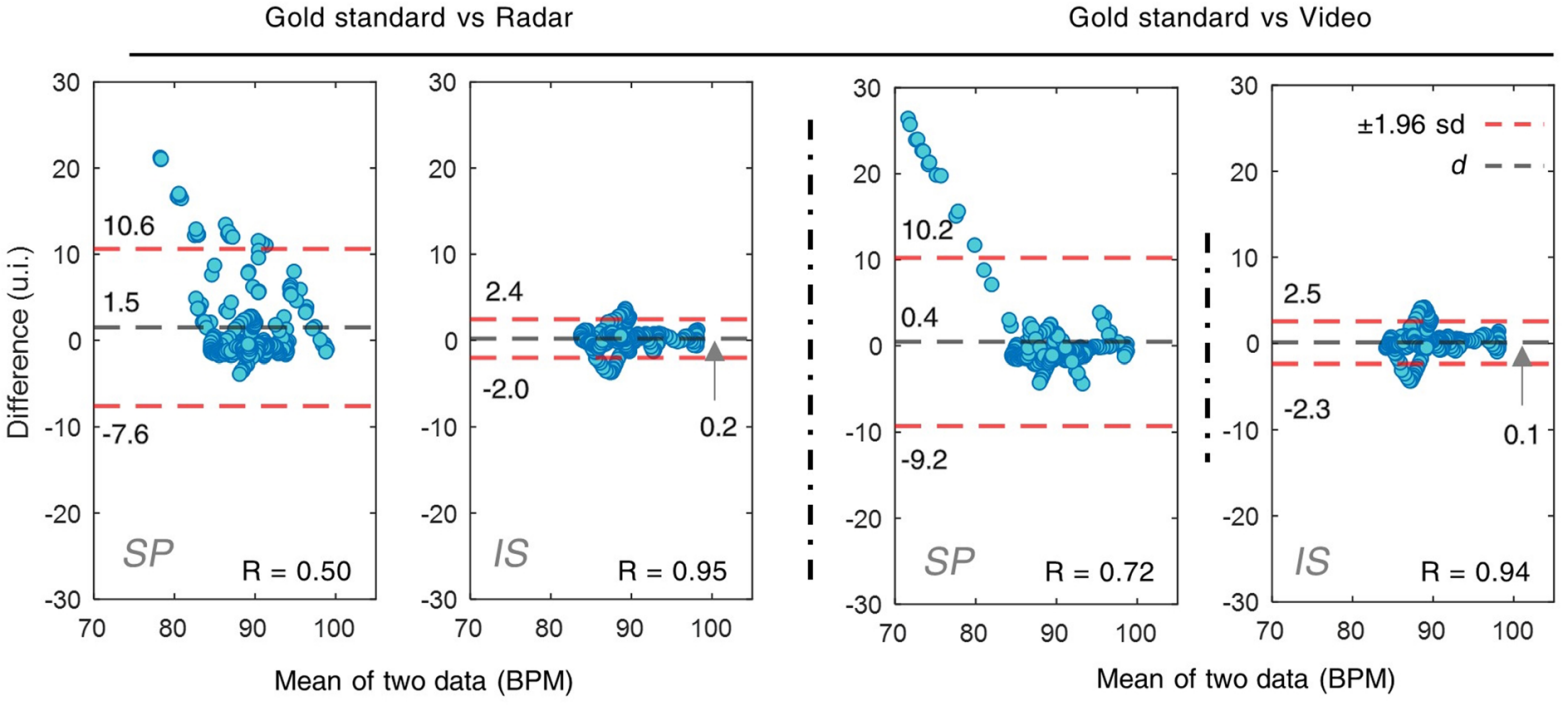
The Bland–Altman plots of the dataset for cross-comparison. SP: results of the spectral magnitude estimation method. IS: results of the image segmentation estimation method. Gold standard: a contact-based ECG device. ± 1.96 sd: the standard deviation of 95% prediction interval. d: the mean of the differences of the data w.r.t the time trace.

The performance of the proposed algorithm is also statistically evaluated by analysing the instantaneous values of the data and the gold standard. The data is presented by using the Bland–Altman plot, as shown in Fig. 8. The Y-axis indicates the difference between the two data, while X-axis indicates the mean of two data. The 95% prediction interval (PI), i.e., the ± 1.96 standard deviation (SD), is the region between the two red dashed lines. The black dashed line is the mean of the difference between the two data. The values of these boundaries are marked on the side of the dashed lines. The correlation coefficients are also placed inside each plot. The data agreement can be evaluated by the range of the PI boundaries and scattering of the data points.

The PI regions of the difference have seen a near 4-time decrease from the conventional to the proposed method. Whereas the mean differences, namely the biases, are also reduced to a negligible level. Intuitively, the data from the proposed method is also less scattered outside of the PI region compared to the spectral magnitude method. The PCC annotation demonstrates 20–40% increases in values and reduced standard deviations of the difference when comparing the proposed method to the conventional one. To conclude, this set of data validation shows that the proposed method achieved around 95% PCCs with a high agreement and negligible bias. A significant performance improvement is observed compared to the conventional method.

### Repeatability test

**Figure 9:**
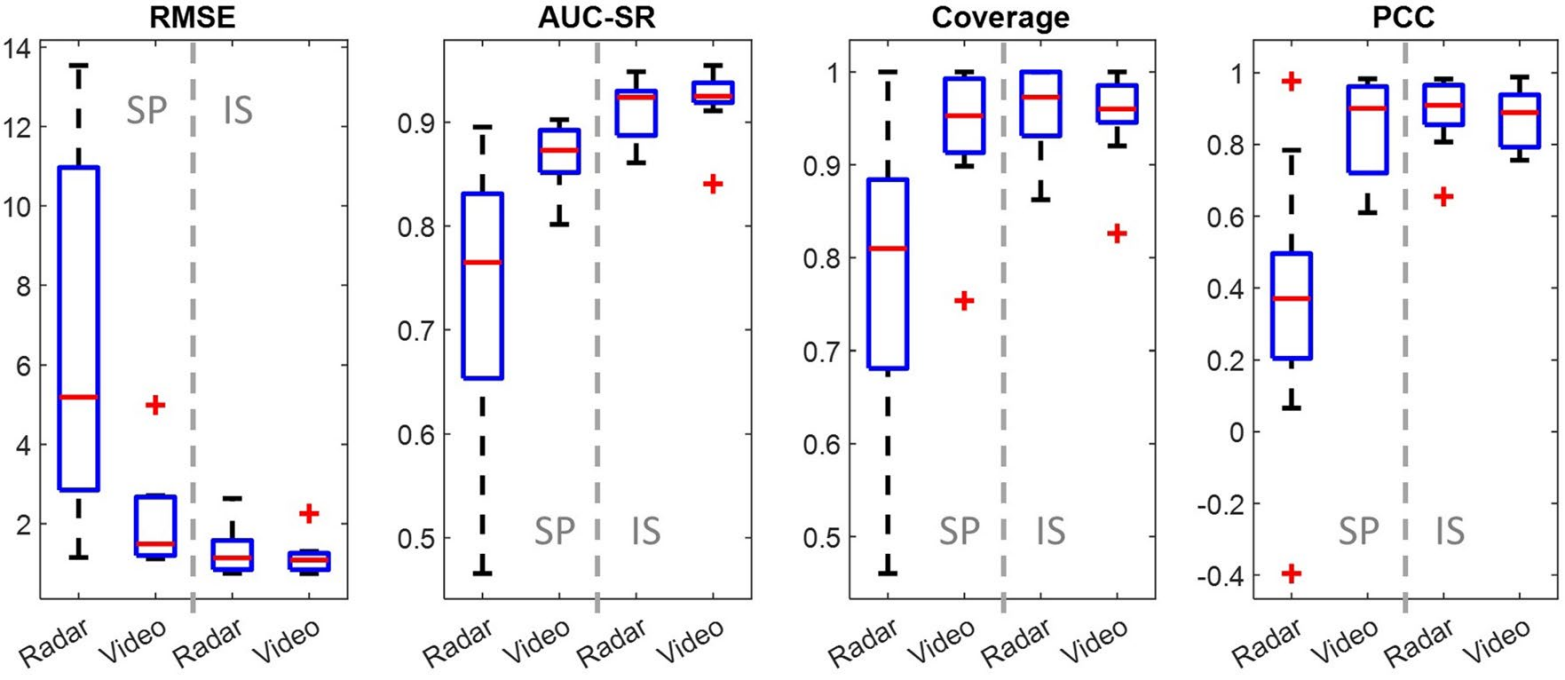
The performance comparison of the conventional and the proposed estimation methods. The boxes on the SP side represent the evaluations of the spectral magnitude method, IS represents the image segmentation method. The median values are indicated by red horizontal bars inside the boxes, the 25 and 75% quartile range by boxes, and the full range by whiskers. Red plus marks represent the outliers.

The boxplots in Fig. 9 are formed by ten datasets, including both the pre- and post-exercise measurements. The left side of each plot represents the evaluation of the spectral magnitude method, whereas the right side represents the proposed method. It can be observed that the range and median values of RMSE are significantly reduced, whereas the AUC-SR, coverage and PCC values are increased with also reduced range. The image segmentation method demonstrates a much-improved estimation accuracy and stability.

Cross-sessional and cross-subject test results of 2-min data are presented in Table 1. The PCCs are also used to indicate the accuracy of the proposed system. The proposed estimation method yields PCCs ranging from 90 to 99% across three subjects in two different sessions, with an average of 94%. The ability to generalise test results is vital to future implementations in practical and more complex scenarios.

**Table 1:** Cross-subject and cross-sessional PCC results.

	Radar vs ECG		Video vs ECG	
	Session 1	Session 2	Session 1	Session 2
Subject 1	0.95	0.97	0.94	0.98
Subject 2	0.93	0.90	0.91	0.91
Subject 3	0.89	0.99	0.90	0.99

### Comparative analysis

The method proposed in this study has been compared with the existing ones to demonstrate the performance. As most of the literature presented self-reported results with various evaluation metrics, the outcome of the comparison is not conclusive. The representative studies of each method, their PCC values, and the conditions in which the experiments were conducted, are selected and listed in Table 2.

**Table 2:** Cross-reference performance comparison.

	Literature	PCC	Method	Experiment conditions
Face video	Poh et al.^34^	95%	Peak detection	Room, ambient lighting condition, sitting still, 12 participants, 1 min
	Li et al.^32^	99%	Spectral magnitude	Room, ambient lighting condition, sitting still, 10 participants, 30 s
	Wang et al.^17^	46%	Deep learning	Room, complex lighting condition, 1–30 min, sitting still
	Ours	94%	Image segmentation	Room, complex lighting condition, sitting still pre&post exercise, 1 participant 10 sessions 5 min, or 3 participants 3 sessions 2 min
FMCW	Alizadeh et al.^6^	80%	Spectral magnitude	Room, lying down, 1 participant, 1 session, 40 min
	Zhao et al.^8^	93%	Deep learning	Room, sitting still, 2 participants, multiple sessions,10 s
	Ours	94%	Image segmentation	Room, sitting still pre&post exercise, 1 participant 10 sessions 5 min, or 3 participants 3 sessions 2 min

From Table 2, multiple studies used a large dataset to demonstrate the stable performance of vital sign estimation produced by the face video-based methods. However, the results from Poh et al.^34^ and Li et al.^32^ were obtained in a highly controlled environment with short testing durations. Improvements need to be made to the deep learning method reported by Wang et al.^17^. The performance of FMCW studies is less comparable as the datasets lack consistency and variety. A shared disadvantage of the existing methods is that they are designed specifically for data from a single source, which means they cannot be used to extract data from face video and FMCW interchangeably.

Our method provides a stable performance on both video and FMCW based estimation with a reasonably sized dataset and in more complex environments. As the vital sign data from both sources can be extracted by the same image segmentation algorithm, our method presented a direction for standardising remote vital sign estimation.

## Conclusions

In this work, we introduced the first use of a graph-based image segmentation algorithm in remote measurements of vital signs. The method performs the segmentation on the STFT spectrograms, which are formed using 20-s data segments from either an FMCW radar or a camera. The method searches for the shortest path across the STFT spectrograms to segment the image along with the layered structures. The found path represents the HR or BR estimation over the duration of a measurement. The experiment was conducted in a lab environment and ten sets of 5-min data were collected for performance evaluation. Compared to the conventional spectral magnitude method, the proposed image segmentation method achieves a significantly improved result over the four evaluation metrics. The image segmentation can also be performed on a rolling basis on the spectrograms for real-time monitoring applications. The experiment was repeated on three different subjects in two sessions, yielding PCCs in the range of 90–98%. The stability, accuracy and adaptability of our method will contribute to the development of contactless telehealth systems, especially under the challenges imposed by Covid-19. This work can be extended to the simultaneous monitoring of multiple individuals in more complex environments in the future.
